# Analyte Recovery of Volatile Organic Compounds: A Passive Sampling Analysis via Photothermal Desorption Compatible Diffusive Samplers

**DOI:** 10.1021/acsomega.5c09208

**Published:** 2026-01-13

**Authors:** Jacob S. Shedd, Evan L. Floyd, Jonghwa Oh, Brie M. McMahan, Claudiu T. Lungu

**Affiliations:** † Department of Environmental Health Sciences, University of Alabama at Birmingham, Birmingham, Alabama 35294-0022, United States; ‡ 34501 SafeBridge Consultants, Inc, Mountain View, California 94043-2503, United States; § Department of Occupational and Environmental Health, 6186 University of Oklahoma, Oklahoma City, Oklahoma 73104, United States

## Abstract

Volatile organic compounds (VOCs) are common sources of occupational exposure throughout a variety of industries. To protect personnel from overexposure, field industrial hygienists must conduct compliance sampling. In efforts to improve upon analytical sensitivity and time-to-knowledge of existing VOC exposure assessment methods, the industrial hygiene research group at UAB has developed a preanalytical technique known as photothermal desorption (PTD), which uses pulses of high-energy light to desorb analytes from thermally conductive, carbonaceous sorbents. To-date, the theoretical and conceptual groundwork for PTD have been laid, and advances have been made toward a first-generation, PTD-compatible diffusive sampler. However, additional characterizations of the prototype sampler’s performance are needed before the method is ready for in-field deployment. As such, the objectives of this study were 2-fold: (1) the primary objective was to determine the percent mass recovered via PTD of samples collected for various VOC analytes (i.e., toluene, *n*-hexane, isopropyl alcohol, and trichloroethylene), and (2) the secondary objective was to quantify the analyte adsorption capacities of buckypaper (BP) sorbents for each VOC of interest. The percent mass recovery of toluene, *n*-hexane, trichloroethylene, and isopropyl alcohol were found to be 0.60 ± 0.09, 1.2 ± 0.09, 1.1 ± 0.1, and 14.0 ± 1.0% per PTD pulse, and analyte adsorption capacities for BP sorbents were determined to be 152 ± 5 mg/g at 219 ppm toluene, 75 ± 42 mg/g at 292 ppm *n*-hexane, 104 ± 37 mg/g at 101 ppm trichloroethylene, and 105 ± 19 mg/g at 413 ppm isopropyl alcohol. The observed differences in desorption of analytes are likely attributed to varying types of weak intermolecular forces acting on aromatic rings, aliphatic chains, and polar moieties. While the large standard deviations in adsorption capacities may be explained by nonuniformity of nanotube alignment in respective sorbents. The early stage, prototype characterization data presented in this study, demonstrates the promising nature of PTD used with passive air samplers and provides a solid foundation for future development of the preanalytical technique and accompanying sampling devices.

## Graphical abstract

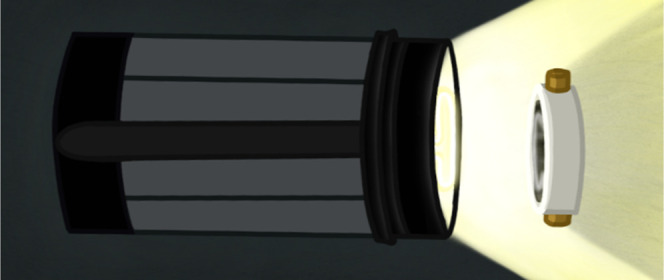

## 1 Introduction

In workplaces across the globe, volatile organic compounds (VOCs) pose an active threat to human health due to their use in a multitude of activities (e.g., industrial, household, agricultural, transportation, etc.). Exposures to VOCs have been reported to cause a plethora of adverse health effects ranging from mucosal irritation,^1^ to organ/systemic damage (i.e., kidney, liver, and central nervous system) and even death.^1–3^

Considering millions of personnel which are employed in occupations known to use VOC-based products,^4^ the use of validated methods^5–8^ of VOC exposure assessment is common among occupational hygienists for the purposes of compliance sampling to comply with the occupational exposure limits from governmental^9^ and professional regulatory bodies.^10,11^ Occupational and industrial hygienists have traditionally used battery-operated sampling pumps with in-line sorbent tubes to collect air samples for analysis. More recently, however, diffusive sampling has become a suitable substitute to the traditional methods of sample collection. The compact, lightweight design of diffusive samplers is regarded as more convenient and comfortable for in-field usage, as opposed to the bulky pumps and motion-restrictive tubing used for active sampling. Unfortunately, the convenience of diffusive samplers comes at the cost of decreased analytical sensitivity, as diffusion-driven mass uptake rate is decreased compared to active sampling methods. The diffusively collected sample mass is further reduced by 200–1000*x* by dilution during prescribed, preanalytical workups also known as chemical desorption (CD)^5–8^ prior to quantification via gas chromatography (GC). These combined sensitivity limitations ultimately result in diffusive sampling being an inadequate method for sample collection over short time durations and in low concentration situations.

In an attempt to address the present limitations of diffusive sampling for short duration or low concentration exposures, our research team developed a preanalytical method for diffusive sampling known as photothermal desorption (PTD), which uses high-energy, visible light to desorb VOCs collected through diffusive sampling from carbonaceous sorbents.^12–16^ PTD’s unique method of desorption eliminates the need for sample extraction and dilution, as prescribed by current analytical methods,^5–8^ while offering repeat desorption cycles and upward of 8 times the sample mass recovery and delivery (i.e., 0.8% per desorption cycle)^16^ of CD with a single GC injection. This is achieved through the use of buckypapers (BP) fabricated from single-walled carbon nanotubes (SWNTs)^12,17,18^ as a thermally conductive sorbent for VOC collection. Following the inception of PTD,^12^ the thermal, surface area, and adsorption characteristics^15,17,18^ of BPs as PTD sorbents have been investigated. Moreover, a study by our research group^19,20^ demonstrated the ability of toluene, *n*-hexane, trichloroethylene (TCE), and isopropyl alcohol (IPA) to be collected by a PTD-compatible diffusive sampler. However, toluene has been the only analyte of interest quantified through PTD studies to-date.^12–14^ As such, the abilities of PTD to recover a variety of VOC analytes must be examined.^19–21^

To address this gap,^19,20^ the objectives of the present work were 3-fold: (1) determine the percent mass recovered via PTD of samples collected for various VOC analytes (i.e., toluene, *n*-hexane, isopropyl alcohol, and trichloroethylene), (2) calculate the prototype’s theoretical sampling rates for each respective analyte, and (3) quantify the analyte adsorption capacity of BP sorbents for each VOC of interest.^22–25^ In doing so, this study is the first to use aa field-ready, prototype, diffusive sampler in conjunction with PTD.

## 2 Methods

### 2.1 Buckypaper Fabrication

SWNTs (arc-discharge, 94.5% pure, 1.2–1.7 nm diameter, 0.1–4 μm length, presuspended in 1% (w/v) sodium cholate or sodium dodecyl sulfate) were obtained from Nanointegris Inc. (Quebec, Canada), and used as received, to fabricate self-supporting BPs for use as sorbent media. The fabrication process used in the present study has been described at length in previous studies by Oh et al.^13,17,18^ and Shedd et al.^15,17–20,25–31^

### 2.2 Equipment Calibration

#### 2.2.1 Photoionization Detector Calibration

Baseline-Mocon 10.6 eV photoionization detectors (PID; VOC-Traq II; Lyons, CO) were calibrated weekly via two-point calibrations with a dry N_2_ (g) zero gas and a span gas consisting of a 100 ppm isobutylene in N_2_ (g) mixture following manufacturer guidelines.

#### 2.2.2 Photothermal Desorption (PTD) Unit Calibration

Adopting a method similar to that of Floyd,^27^ analyte calibration curves (12-point) were generated periodically for the entire PTD system by injecting a range of vaporous masses of toluene (1.28–44.93 μg; *n* = 12; ACS grade), *n*-hexane (1.29–1030 μg; *n* = 18; 99+% purity), trichloroethylene (TCE; 1.40–44.23 μg; *n* = 12; 99.9% purity), and isopropyl alcohol (IPA; 27.98–736.19 μg; *n* = 12; 99.9% purity). VOC-Traq II software (v. 1.0.0.32; Baseline-Mocon; Lyons, CO) was used to monitor and log calibration data in real-time. The concentration data were then corrected for each analyte using manufacturer provided PID response factors. Manual peak-picking was performed on the corrected data, and the selected curves were integrated from baseline-to-baseline to quantify the analyte mass detected. The regression information for each analyte calibration curve has been reported in Table 1.

**Table 1:** Analyte Calibration Equations and Fit (*r* ^2^)­^a^

analyte	calibration equation	*r* ^2^
toluene	*m*_*d*_ = 0.414099*m*_*i*_ – 1.66844	0.9301
*n*-hexane	*m*_*d*_ = 0.279259*m*_*i*_ + 1.198642	0.9865
trichloroethylene (TCE)	*m*_*d*_ = 0.295711*m*_*i*_ – 1.61877	0.9335
isopropyl alcohol (IPA)	*m*_*d*_ = 0.213206*m*_*i*_ + 8.94656	0.9946

a Where mi is mass injected (μg) and md in mass detected (μg).

### 2.3 Design of Prototype Sampler

First-generation prototype samplers (Figure 1), as designed by Shedd et al.,^19,20^ were drafted by Creative Engineering (Bronxville, NY), using AutoCAD (Autodesk; San Rafael, CA), and manufactured from nylon-6,6 via computer numerical control machining (manufacturing partner: Xometry, Gaithersburg, MD). A two-sided sampler design was chosen for the prototypes to allow for adsorption and desorption via PTD on the opposing sides of the sampler body. When used in sampling, analytes penetrate through the stainless-steel mesh windscreen (pore diameter: 0.014 cm; open area: 30%; McMaster-Carr; Elmhurst, IL) seen in Figure 1a, via downfield diffusion and are adsorbed onto a BP housed on the opposite side of the sampler. On the reverse side of the sampler (Figure 1b), a BP is positioned against a quartz window (diameter: 3.81 cm; thickness: 0.32 cm; McMasterCarr; Elmhurst, IL), allowing for light to irradiate the BP during PTD. Two brass fittings (0.32 inner diameter) were also affixed to the sampler body (Figure 1) to allow for the passage of N2 (g) through the sampler for instrumental analysis. Caps were securely fastened to the fittings during analyte sampling to close the system and prevent disturbances from occurring in the diffusion gradient.

**Figure 1:**
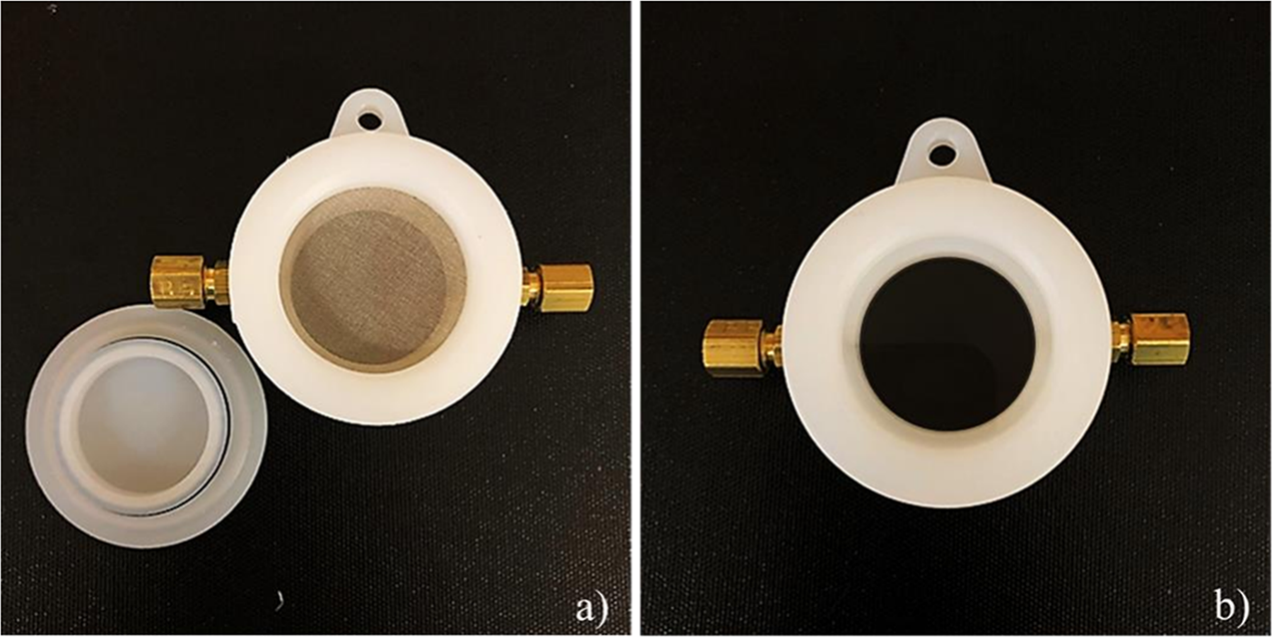
Alternate sides of a PTD-compatible diffusive sampler. (1a). Depicts a diffusion side-up view of the sampler with the diffusion cap sitting next to the sampler body. (1b). Depicts the desorption sideup view of the sampler, with a BP loaded below the quarts window.

### 2.4 Dynamic Exposure Chamber

Sampler dosing (*n* = 4 per dosing) was performed using the 2.5 L (1152 in^3^) aluminum exposure chamber described Shedd et al.^19,20^ A constant air flow was provided to the chamber by a 96 L air compressor (Kobalt Quiet Tech; Lowe’s Companies, Inc.; Mooresville, NC), and a flow rate (Q) of 74 L/min was maintained by an in-line mass-flow controller (range: 0–100 SLPM; Sierra Instruments; Monterey, CA), based on a applied inlet pressure of approximately 827.4 kPA (Figure 2). The compressed air was dried by flowing through an anhydrous CaSO_4_ desiccant and water trap prior to entering the mass-flow controller, and analytes of interest were introduced into the air stream via programmable syringe pump (Model: F100X; Chemyx; Stafford, TX), through an injection port located directly after the mass-flow controller. Analyte and air mixing was promoted by the presence of a stainless-steel mixing mesh (open area: 22%), which also served to reduce the chamber’s cross-sectional area to 90.8 cm^2^ and allowed for the generation of sampler face velocities of 26.5 ft/min.

**Figure 2:**
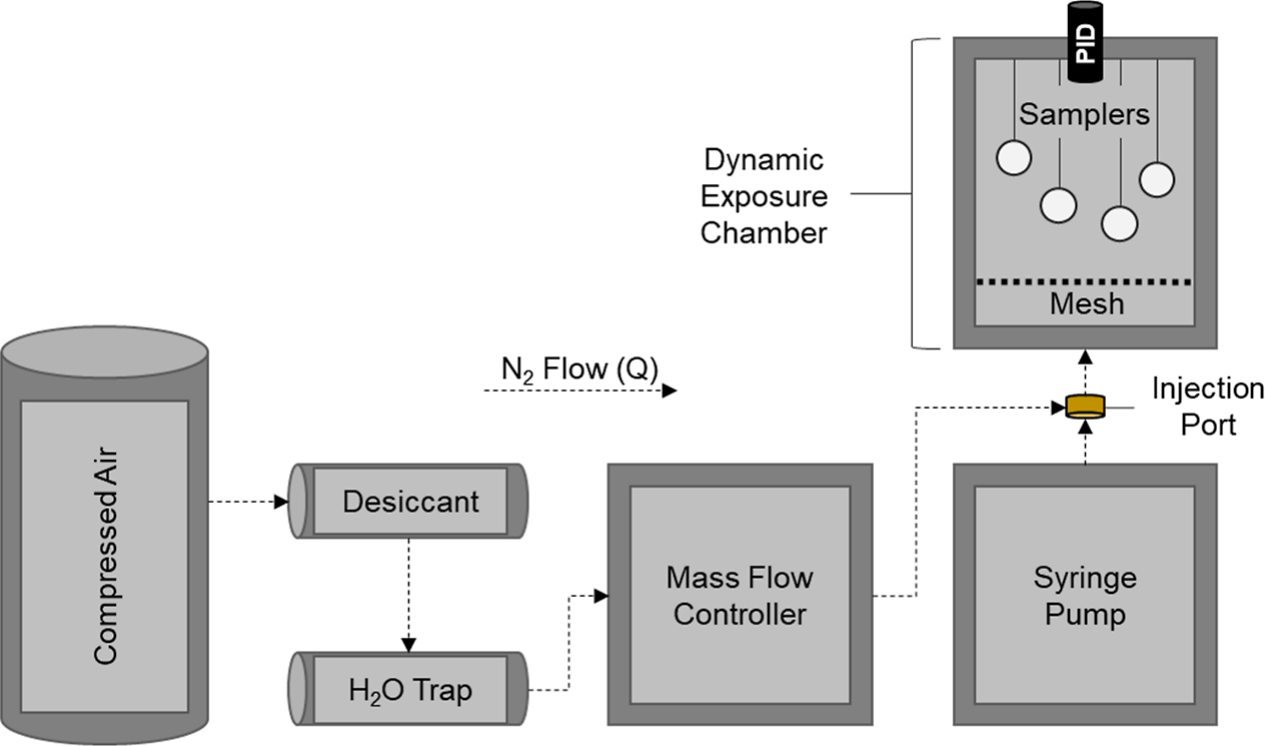
Simplified flow diagram of dynamic exposure chamber construction.

For the collection of each respective analyte, four prototype samplers were hung at randomized heights (i.e., 18.7, 21.4, 25.4, and 27.5 cm) within the exposure chamber (Figure 2) and positioned so that their diffusion barriers were facing inward. Samplers were exposed to concentrations near the permissible exposure limits (PELs) set by the Occupational Safety and Health Administration (OSHA) for each analyte of interest, respectively (toluene: 200 ppm, *n*-hexane: 200 ppm, TCE: 100 ppm, IPA: 400 ppm).^25,28–30^ Analyte concentrations were simultaneously verified via an in situ PID suspended at a depth of 25.4 cm within the chamber. At the conclusion of each sample collection run, the samplers were capped with form-fitting, nylon-6,6 covers over their diffusion barriers, completely wrapped in a blended wax and polyolefin film, and stored in a 5 °C refrigerator (storage time: 24–72 h) to maintain sample integrity prior to desorption via PTD.

### 2.5 Mass Recovery by Photothermal Desorption

Exposed, capped samplers were connected to a PTD unit and in-line PID as seen in Figure 3. Each sampler was pulsed 4 times with visible light (energy density: 1.33 J/cm^2^ as reported by Shedd et al.^15^) from the PTD unit’s xenon flash lamp (Neewer 300 W, Neewer Technology, Shenzhen Guangdong, China), taking special care to let the system purge between each desorption cycle (i.e., light pulse). The resulting desorption curves were corrected for isobutylene equivalents using response factors provided by the PID manufacturer^31^ for each analyte of interest and approximately integrated using left-aligned Riemann sums. The sums were then averaged for each respective sampler to determine the average mass recovered per desorption cycle. The percentage of the sampled mass recovered by PTD (%_recovered_) was subsequently determined by dividing the average mass recovered by the theoretical mass sampled based on the sampling rates reported in our previous work by Shedd et al.^19^ (Table 2).

**Figure 3:**
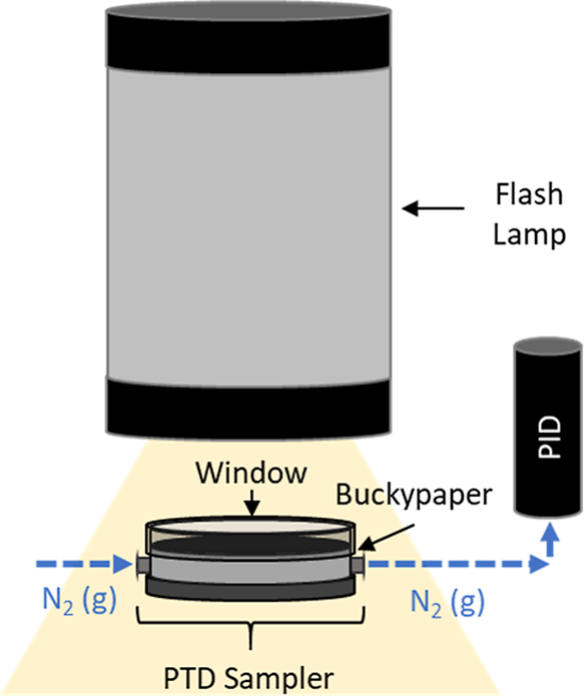
Diagram of photothermal desorption unit, illustrating sampler irradiation under N_2_ (g) flow.

**Table 2:** Sampling/Uptake Rates of PTD-Compatible Diffusive Samplers^20^

analyte	UR_Ideal_ (cm^3^/min)	UR_Eff_ ^a^ ^,^ ^b^ (cm^3^/min)
toluene	12.96	28 ± 2
*n*-hexane	12.32	21 ± 1
trichloroethylene (TCE)	13.24	15 ± 1
isopropyl alcohol (IPA)	16.47	8.5 ± 0.7

a Values written as “average ± error”.

b *n* = 4. UR_ideal_ and UR_eff_ are the theoretically and empirically determined uptake rates of PTD-compatible samplers, respectively. Uptake rates listed are reprinted in part with permission from Shedd, J. S.; Oh, J.; Floyd, E. L.; Lungu, C. T. Characterization of Photothermal Desorption-Compatible Diffusive Samplers for VOCs. ACS Environmental Au. 2023, 3 (4), 242.^19^

### 2.6 Analyte Adsorption Capacity

Adsorption isotherms and BP adsorption capacities were determined for each analyte of interest using a diffusive adsorption isotherm chamber (DAIC) previously designed by our research group.^17^ To do so, a vial of respective challenge analyte was connected to the DAIC as seen in Figure 4 and allowed to diffuse into the chamber at a constant temperature of 30 °C (303.15 K). Increasing analyte concentrations within the chamber were monitored in real-time via an in situ 10.6 eV PID (Figure 3; VOC-Traq II; Baseline-Mocon; Lyons, CO). Concentration data was converted to mass present in the chamber via eq 1.^17^ and used to quantify the mass flux of each given VOC; changes in the mass present in the chamber was used to quantify the mass flux of each given VOC over a continuum of chamber concentrations when held at the constant temperature.

1 $$\Delta m=\left(\frac{\Delta C}{V_{m}}\times M_{W}\right)\times V_{C}\times 10^{3}$$

where Δ*m* is change in mass of analyte at a given time interval (μg), Δ*C* is the change in VOC concentration within the chamber at a given time, *V*_*m*_ is the molar volume of an ideal gas (25.04 m^3^/mol at standard ambient temperature and pressure; SATP),^17^ *M*_W_ is the analyte molecular weight (g/mol), and V_C_ is the volume of the diffusion chamber (4.73 × 10^–5^ m^3^).

**Figure 4:**
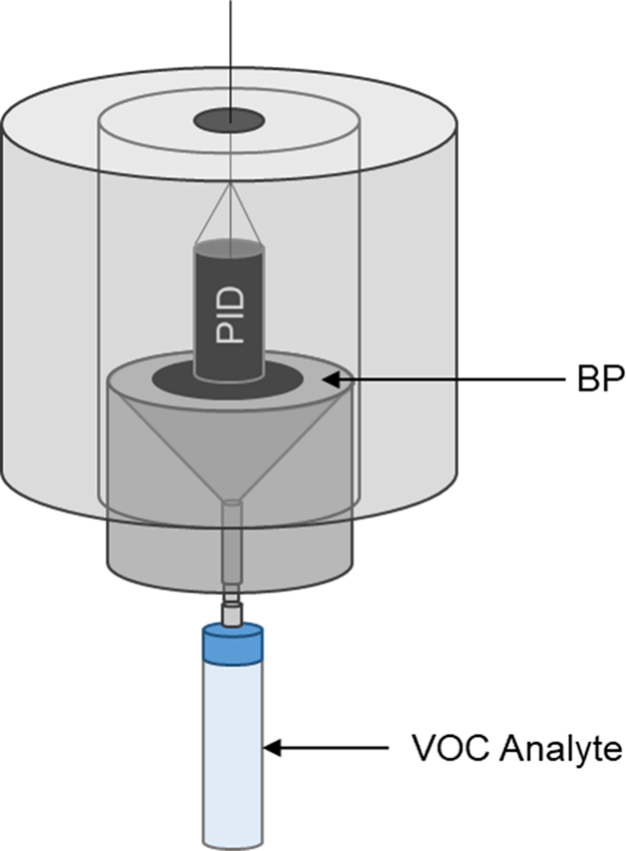
Diagram of DAIC depicting the diffusion of analyte onto a BP housed within the chamber.

Respective adsorption isotherms were collected in triplicate with pristine BP samples up to 600 ppm for each analyte and expressed in a plot as mg of adsorbed VOC per gram of BP mass. Adsorption capacities for the AD BPs were calculated at the OSHA PEL for each analyte (toluene, *n*-hexane, TCE, and IPA)^22–25^ using the adsorption isotherm data.

### 2.7 Statistics

Dixon’s Q-test^32^ was used for the detection of outliers within the data (α = 0.05) and compared to a table of critical *Q*-values (*Q*_crit_).^33^

## 3 Results

### 3.1 Theoretical Sampling Rates and Sample Mass Recovery

Table 3 describes the average, real-time exposure data within the exposure chamber, the calculated sampling rates for the prototype samplers, and the average analyte masses recovered via PTD (*n* = 4 for Toluene, TCE, and IPA; *n* = 3 for *n*-hexane). Data from the in situ, chamber PID was observed to be near the PELs for each respective analyte. Sampling rates for each analyte were assumed based on the empirical findings reported by Shedd et al.,^19^ and resulted in sample masses being collected in the range of 4.0 to 11.0 mg per 8 h sampling period, depending on the analyte being sampled (Table 3). Once flashed with a single strobe from the PTD unit, average sample masses recovered for toluene, *n*-hexane, TCE, and IPA were observed to be 0.031 ± 0.002, 0.075 ± 0.006, 0.038 ± 0.003, and 1.15 ± 0.02 mg respectively, resulting in percent sample masses recovered per single PTD flash ranging between 0.27 ± 0.04 and 27.8 ± 2.3%.

**Table 3:** Sampling Rate and Characterization of Sampled Analyte Mass Recovered by PTD

	PID		sampling rate^c^ ^20^	%_Recovered_ perPTD flash		
analyte	time (h)	concentration (ppm)^a^	cm^3^/min	m_sampled_ (mg)^b^	m_Recovered_ ^b^(mg)	%_Recovered_ ^b^
toluene	8.05	219 ± 57	28.0 ± 2.0	11.0 ± 3.0	0.031 ± 0.002	0.27 ± 0.04
*n*-hexane	8.02	292 ± 43	21.0 ± 1.0	10.0 ± 2.0	0.075 ± 0.008	0.7 ± 0.1
TCE	8.00	101 ± 18	15.0 ± 1.0	4.0 ± 0.8	0.038 ± 0.003	1.0 ± 0.1
IPA	8	413 ± 56	8.5 ± 0.7	4.1 ± 0.7	1.15 ± 0.02	27.8 ± 2.3

a Values are depicted as average ± STDEV.

b Values are depicted as average ± error.

c Sampling rates listed reprinted in part with permission from Shedd, J. S.; Oh, J.; Floyd, E. L.; Lungu, C. T. Characterization of Photothermal Desorption-Compatible Diffusive Samplers for VOCs. ACS Environmental Au. 2023, 3 (4), 242.^19^

Conducting Dixon’s Q-test^32^ on the raw mass data recovered via PTD from samplers 1–4 failed to reveal the presence of significant outliers. However, conducting the Q-test^32^ on the averaged data of individual samplers, after correcting with the corresponding calibration regression, highlighted the average *n*-hexane data collected by sampler 4 as a significant outlier (*Q* = 0.972; *Q*_crit_ = 0.829^40^; *n* = 4; α = 0.05), prompting the removal of the data from further processing. Including sampler 4 data resulted in *n*-hexane m_Recovered_ and %_Recovered_ values of 0.153 ± 0.007 mg and 2.5 ± 0.3%, respectively.

### 3.2 Analyte Adsorption Isotherms and Capacities

Triplicate adsorption isotherms were conducted for toluene, *n*-hexane, TCE, and IPA, with Figure 5 representing the respective isotherm data collected from a single BP sample. Average adsorption capacities for each analyte at near-PEL concentrations (i.e., the concentrations obtained within the exposure chamber during dosing) were calculated based on the observed adsorption isotherms and are presented in Table 4.

**Figure 5:**
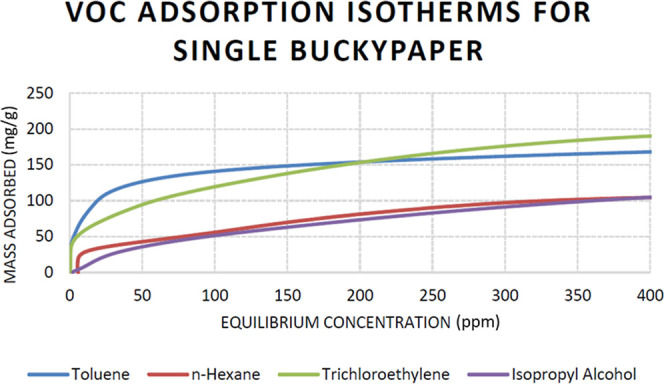
Representative VOC adsorption isotherms for a single buckypaper respectively dosed with toluene, *n*-hexane, TCE, and IPA.

**Table 4:** Adsorption Capacities for Analytes of Interest at Concentration Achieved in the Sampling Chamber

analyte	concentration (ppm)	adsorption capacity (mg/g)^a^	adsorption capacity per BP (mg)^b^
toluene	219	152 ± 5	7.6 ± 0.3
*n*-hexane	292	75 ± 42	4.0 ± 2.0
trichloroethylene (TCE)	101	104 ± 37	5.0 ± 2.0
isopropyl alcohol (IPA)	413	105 ± 19	5.0 ± 1.0

a Values depicted as average ± STDEV, *n* = 3.

b BP mass: 50 mg.

## 4 Discussion

As mentioned in the Introduction section of the present work, current analytical methods for CD result in the GC analysis of only 0.01 to 0.2% of a collected sample mass.^5–8^ Based on the results presented in Table 2, this study has demonstrated that the use of PTD in tandem with a PTD-compatible diffusive sampler can increase the percent of desorbed analyte available for analysis. Specifically, the percent of sample mass recovered per PTD flash ranged from 0.27 ± 0.04 to 27.8 ± 2.3%, depending on the analyte. This means that the integration of PTD with a compatible diffusive sampler has resulted in an increased sample recovery of 1.35 to 139*x* that provided by the highest sample mass produced from traditional GC methods (i.e., 0.2%), thus enabling the delivery of higher mass aliquots to downstream instrumentation. Furthermore, the percent of toluene desorbed by PTD in this study is on par with the mass recovered from similar PTD studies by Floyd et al. and Oh et al. which ranged from 0.001–0.86% desorption, depending on the PTD energy input.^13,16^ This comparability to the literature stands to validate this method as consistent and reproducible. In addition, the data recovered from the PTD of each analyte was noted to be fairly consistent when grouped by analyte of interest, as evidenced by the small standard deviations in %_Recovered_ data reported in Table 3, further validating the method for analytes beyond toluene. That said, the large percentage of recovered IPA mass does appear to be off, considering the sorbent was not exhausted of analyte within 4 subsequent flashes. This indicates the potential for under-reporting of the IPA sampling rate. Since the sampling rate of IPA was determined from empirical data,^19^ this underreporting likely resulted from the limited compatibility of IPA with the low-polarity hydrocarbon GC column used during sample analysis. As such, it may be worth considering the mass collected and recovered that were calculated from the theoretical uptake rate of IPA (16.47 cm^3^/min) reported in our previous work.^19^ Based on this value, the total mass of IPA sampled (*m*_sampled_) would be 8.0 ± 1.0 mg, and the percent of IPA mass recovered by PTD (%_Recovered_) would be 14.0 ± 1.0%. This means rather than delivering 139x more mass than a 0.2% (m/m) injection into a GC, IPA might have mass delivery closer to 70*x*. Future studies on PTD-compatible samplers should consider validating the IPA uptake rate using a more appropriate GC column.

It is worth noting that after the collection of analytes via prototype sampler, off-gassing from sampled BPs was detected upon connecting the prototype with the PTD system. This had the effect of high baseline concentrations that needed to be purged prior to conducting analyte desorption. To determine if the observed off-gassing was the result of oversaturating the BP sorbent, analyte adsorption capacities were measured for each analyte of interest at the same concentrations used to dose the prototypes in the sampling chamber (Table 5). From the data presented in Table 5, it is evident that BPs were potentially oversaturated with Toluene and *n*-hexane. However, it is interesting that the TCE and IPA masses collected were less than their respective adsorption capacities considering there was evidence of off-gassing from all analytes. Given this information, it is possible that other factors are at play in the observed off-gassing of samples. Off-gassing in TCE and IPA samples may be the result of analyte desorbing from the nylon 6,6 sampler housing. This might also be an additional factor in sorbent off-gassing in the case of toluene and *n*-hexane oversaturation. Regarding the reported adsorption data, in general, it should be noted that large deviations were observed from one BP to the next (Tables 4 and 5). This can most likely be attributed to the methods used for BP fabrication, which does not uniformly align nanotubes during fabrication. This means that the adsorption sites from one BP to the next are similar, but never identical. Going forward, future studies should consider adopting fabrication techniques that enable increased control of nanotube alignment. It may also be worth considering the use of functionalized sorbents to increase the adsorption affinity for given analytes.

**Table 5:** Comparative Values for Analyte Adsorption Capacity and Empirically Sampled Analyte Mass

analyte	concentration (ppm)	adsorption capacity per BP (mg)^a^	*m*_sampled_ (mg)^b^
toluene	219	7.6 ± 0.3	11.0 ± 3.0
*n*-hexane	292	4.0 ± 2.0	10.0 ± 2.0
trichloroethylene (TCE)	101	5.0 ± 2.0	4.0 ± 0.8
isopropyl alcohol (IPA)	413	5.0 ± 1.0	4.1 ± 0.7

a Values depicted as average ± STDEV, *n* = 3.

b BP mass: 50 mg.

Though painstaking steps were taken to reduce systemic errors wherever possible, this study is not without its limitations. In particular, the prototype samplers developed in the present work were noted to experience potential oversaturation of analyte during dosing, and analyte readsorption during PTD. The potential for oversaturation was hinted at by the high, baseline concentrations of analyte detected by the PID immediately after attaching a prototype sampler to the PTD system, indicating analyte off-gassing from the BP sorbent. Oversaturation was further evidenced by the sample masses (*m*_sampled_) of toluene and *n*-hexane being higher than their respective adsorption capacities. However, the analyte adsorption capacities of TCE and IPA indicate BPs should be able to adsorb far more than the sampled mass presented in Table 5. The presence of off-gassing in samples of these analytes may be due to desorption occurring from the plastic of the sampler body. That said, it should be noted that data was not collected on the stability of samples held in cold storage over time. Though evidence of oversaturation is present, it cannot be ruled out that analyte loses may have occurred between sample collection and desorption.

In addition to oversaturation, it should be noted that the standard deviations of analyte concentrations within the dynamic sampling chamber were relatively high. This was due to a limitation in the air compression equipment used in the study. While exposing the samplers in the chamber, the air compressor was allowed to run continuously; this led to rises and drops in air pressure as the compressor’s air tank was depleted and recharged. Real-time observations of the PID data (concentration over time) during compressor cycles indicated a drop in analyte concentration within which corresponded to a drop in air pressure as the compressor tank was depleted. The analyte concentration the rebounded to the target concentration as the compressor tank recharged.

In the case of readsorption, the double-sided design of the prototype used in the present work requires analyte adsorption and desorption to occur on opposite sides of the sorbent (Figure 6). This is particularly important to consider as a study by Shedd et al. demonstrated that the direct, radiant heating of a BP surface may not penetrate through the bulk of the material based on temperature data collected at differing BP depths.^15^ Instead, the heat is more likely to experience peak conduction in the plane of the BP surface and not across the thickness of the BP. As such, there is a potential for more analyte to desorb from the irradiated side than from the side of adsorption. While some of the analyte vapor would make its way into the carrier gas, the rest would remain in the submillimeter headspace between the desorption side of the BP and the window (Figure 6). Evidence of readsorption could be inferred from the individual PTD peaks recorded by the PID. Unlike previous PTD studies^12–14,16^ where a gradual exhausting of analyte was seen with sequential desorption cycles (i.e., PTD flashes), analyte peaks from the present work were not observed to follow the established pattern. Instead, subsequent desorption peaks were more randomized.

**Figure 6:**
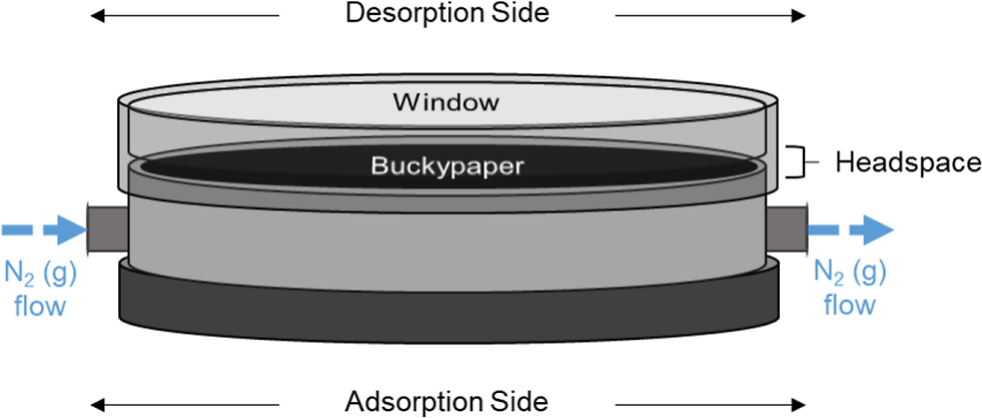
Exaggerated depiction of submillimeter headspace within a PTD-compatible diffusive sampler.

The only cases where the previously established pattern was observed were during experimental runs where the seal between the BP-window headspace was disturbed (i.e., pre-existing cracks in the BP edge or the BP slipping out of place due to thermal shearing at the edges during PTD). Going forward, future studies may want to consider the use of less rigid BPs, so as to be more resistant to cracking and shearing. Additionally, future iterations of PTD-compatible diffusive samplers may benefit from a retooling of the design to allow for adsorption and desorption to occur on the same side of the sorbent.

## Conclusion

The present work sought to develop and characterize the performance of a first-generation prototype diffusive sampler used in conjunction with the PTD technique. This was accomplished by quantifying the sample mass recoverable from the sampler via PTD and measuring the adsorption capacities of the BP sorbents housed within the sampler. This study found that PTD can recover 0.27 ± 0.04, 0.7 ± 0.1, 1.0 ± 0.1, and 27.8 ± 2.3% of the respective toluene, *n*-hexane, TCE, and IPA masses collected via prototype sampler per PTD flash. The present work also quantified the analyte adsorption capacities of BP sorbents to be 152 ± 5 mg/g at 219 ppm of toluene, 75 ± 42 mg/g at 292 ppm of *n*-hexane, 104 ± 37 mg/g at 101 ppm of TCE, and 105 ± 19 mg/g at 413 ppm of IPA. The data presented in the present study demonstrates the viability of using diffusive samplers in tandem with PTD and lays promising groundwork for future developments of this emerging sampling and analysis technology.

## Data Availability

The authors confirm that the data supporting the findings of this study are available within the article. Additional questions concerning the data presented may be addressed to the corresponding author [C.T.L].

## Abbreviations

VOCs: Volatile organic compounds
OELs: pccupation exposure limits
CD: chemical desorption
GC: gas chromatography
PTD: photothermal desorption
BP: Buckypapers
SWNTs: single-walled carbon nanotubes
PID: photoionization detector
CNC: computer numerical control
PEL: permissible exposure limit
DAIC: diffusive adsorption isotherm chamber

## References

[1] ASTDR . INTERACTION PROFILE FOR: Benzene, Toluene, Ethylbenzene, and Xylenes (BTEX); Department of Health and Human Services Public Health Service Agency for Toxic Substances and Disease Registry. 2004. 38569062

[2] WHO Working Group Updating and Revision of the Quality Guidelines for Europe; Report on WHO Working Group on Volatile Organic Compounds: Brussels, Belgium, 1996.

[3] Compendium of Methods for the Determination of Toxic Organic Compounds in Ambient Air; U.S. EPA, 2nd ed., 1999, Cincinnati.

[4] US Bureau of Labor Statistics . Labor force statistics from the current population survey. https://www.bls.gov/cps/lfcharacteristics.htm#occind.

[5] NIOSH . NIOSH Manual of Analytical Methods. Hydrocarbons, BP 36°-216°C (NIOSH-1500) 1994(2), 8–11.

[6] NIOSH . NIOSH Manual of Analytical Methods. Hydrocarbons, Aromatic (NIOSH-1501) 2003, 127(3), 1–7..

[7] NIOSH . NIOSH Manual of Analytical Methods. Hydrocarbons, Halogenated (NIOSH-1003) 2003, 1–7.

[8] Elskamp, C. J. OSHA Sampling and Analytical Methods - Toluene (Organic Method #111) . OSHA Sampling and Analytical Methods, U.S. Department of Labor. https://www.osha.gov/dts/sltc/methods/organic/org111/org111.pdf.

[9] Occupational Safety and Health Administration. 29 CFR 1910. https://www.osha.gov/laws-regs/regulations/standardnumber/1910.

[10] National Institute for Occupational Safety and Health (NIOSH) . NIOSH Pocket Guide to Chemical Hazards. https://www.cdc.gov/niosh/npg/default.html. 11326577

[11] American Conference of Governmental Industrial Hygienists (ACGIH) 2019 TLVs and BEIs; ACGIH Signature Publications: Cincinnati, 2019.

[12] Floyd E. L., Sapag K., Oh J., Lungu C. T. (2014). Photothermal
Desorption
of Single-Walled Carbon Nanotubes and Coconut Shell-Activated Carbons
Using a Continuous Light Source for Application in Air Sampling. Ann. Occup. Hyg..

[13] Oh, J. Fabrication and Characterization of Buckypapers for Use in Air Sampling. Ph.D. Dissertation; University of Alabama at Birmingham, 2016.

[14] Floyd, E. L. Photothermal Desorption of Toluene From Single Walled Carbon Nanotubes and Activated Carbon Sorbents. Ph.D. Dissertation; University of Alabama at Birmingham, 2013.

[15] Shedd J. S., Kuehster W. W., Ranjit S., Hauser A. J., Floyd E. L., Oh J., Lungu C. T. (2021). Determining
the Thermal Properties of Buckypapers Used
in Photothermal Desorption. ACS Omega.

[16] Floyd E. L., Oh J., Sapag K., Oni T. M., Shedd J. S., Lungu C. T. (2022). Photothermal
Desorption of Toluene from Carbonaceous Substrates Using Light Flash. Nanomaterials.

[17] Oh J., Floyd E. L., Watson T. C., Lungu C. T. (2016). Fabrication and
Adsorption Characterization of Single-Walled Carbon Nanotubes (SWNT)
Buckypaper (BP) for Use in Air Samples. Anal.
Methods.

[18] Oh J., Floyd E. L., Parit M., Davis V. A., Lungu C. T. (2016). Heat Treatment
of Buckypaper for Use in Volatile Organic Compounds Sampling. J. Nanomater..

[19] Shedd J. S., Oh J., Floyd E. L., Lungu C. T. (2023). Characterization of Photothermal
Desorption-Compatible Diffusive Samplers for Volatile Organic Compounds. ACS Environmental Au.

[20] Shedd, J. S. Photothermal Desorption of Volatile Organic Compounds From Photothermal Desorption of Volatile Organic Compounds From Buckypaper Sorbents, for Occupational Exposure Assessment. Ph.D. Dissertation; University of Alabama at Birmingham, 2022. https://digitalcommons.library.uab.edu/etd-collection://digitalcommons.library.uab.edu/etd-collection/333.

[21] Jones S. R., Shedd J. S., Oh J., Lungu C. T. (2022). Evaluating the Effects
of Modified Windscreens on Organic Vapor Monitor Performance. Environ. Health Insights.

[22] National Institute for Occupational Safety and Health (NIOSH) . NIOSH Pocket Guide to Chemical Hazards: Toluene. https://www.cdc.gov/niosh/npg/npgd0619.html (accessed Jan 06, 2020).

[23] National Institute for Occupational Safety and Health (NIOSH) . NIOSH Pocket Guide to Chemical Hazards: n-Hexane. https://www.cdc.gov/niosh/npg/npgd0322.html (accessed Jan 06, 2020).

[24] National Institute for Occupational Safety and Health (NIOSH) . NIOSH Pocket Guide to Chemical Hazards: Trichloroethylene. https://www.cdc.gov/niosh/npg/npgd0629.html (accessed Jan 06, 2020).

[25] National Institute for Occupational Safety and Health (NIOSH) . NIOSH Pocket Guide to Chemical Hazards: Isopropyl Alcohol 2019. https://www.cdc.gov/niosh/npg/npgd0359.html.

[26] Shedd J. S., Floyd E. L., Oh J., Lungu C. T. (2019). FTIR Determination
of Surfactant Removal from Arc Discharge Buckypapers for Air Sampling. J. Adv. Nanomater.

[27] Floyd, E. L. Photothermal Deorption of Toluene From Single Walled Carbon Nanotubes and Activated Carbon Sorbents. P.h.D Dissertation; University of Alabama at Birmingham, 2013.

[28] National Institute for Occupational Safety and Health (NIOSH) . NIOSH Pocket Guide to Chemical Hazards: Toluene. https://www.cdc.gov/niosh/npg/npgd0619.html (accessed Jan 05, 2020).

[29] National Institute for Occupational Safety and Health (NIOSH) . NIOSH Pocket Guide to Chemical Hazards: n-Hexane. https://www.cdc.gov/niosh/npg/npgd0322.html (accessed Jan 05, 2020).

[30] National Institute for Occupational Safety and Health (NIOSH) . NIOSH Pocket Guide to Chemical Hazards: Trichloroethylene. https://www.cdc.gov/niosh/npg/npgd0629.html (accessed Jan 05, 2020).

[31] Baseline-Mocon . PiD-TECH® EVx User Manual 143–175; Rev 1.0 2000.

[32] Dean R. B., Dixon W. J. (1951). Simplified Statistics
for Small Numbers of Observations. Anal. Chem..

[33] Harvey, D. Analytical References: Appendix 06: Critical Values for Dixon’s Q-Test. Chemistry LibreTexts. https://chem.libretexts.org/Ancillary_Materials/Reference/Reference_Tables/Analytic_References/Appendix_06%3A_Critical_Values_for_Dixons_Q-Test (accessed August 18, 2022).

